# The Draft Genome Sequence of *Sphingomonas* sp. Strain FukuSWIS1, Obtained from Acidic Lake Grosse Fuchskuhle, Indicates Photoheterotrophy and a Potential for Humic Matter Degradation

**DOI:** 10.1128/genomeA.01183-14

**Published:** 2014-11-13

**Authors:** Ivette Salka, Abhishek Srivastava, Martin Allgaier, Hans-Peter Grossart

**Affiliations:** a Leibniz-Institute of Freshwater Ecology and Inland Fisheries, Stechlin, Germany; b Berlin Center for Genomics in Biodiversity Research, Berlin, Germany; c Potsdam University, Institute for Biochemistry and Biology, Potsdam, Germany

## Abstract

*Sphingomonas* spp. are *Alphaproteobacteria* considered to be versatile bacteria that can utilize a variety of natural substrates available in terrestrial and aquatic systems. *Sphingomonas* sp*.* strain FukuSWIS1 was isolated from the eutrophic and acidic freshwater Lake Grosse Fuchskuhle in northeastern Germany. The strain has a genome size of 3.89 Mb, possesses a set of photosynthetic genes, and expresses photopigment BChl *a* under oxic conditions. Thus, this strain belongs to the aerobic anoxygenic phototrophic (AAP) bacteria, which are most likely involved in humic matter degradation as indicated by the presence of organic compound mineralizing genes.

## GENOME ANNOUNCEMENT

Freshwater ecosystems were recently shown to possess a remarkable number of aerobic anoxygenic phototrophic (AAP) bacteria (1). These are heterotrophic bacteria that possess a photosystem allowing for anoxygenic photosynthesis under aerobic conditions, but simultaneously lack the ability to grow photoautotrophically (2). Several genomes of marine AAP bacteria provide insights into their metabolic capabilities. However, information on phylogenetic diversity and the genomic potential of freshwater AAP bacteria is still missing. A strain belonging to the genus *Sphingomonas* was isolated from the acidic, humic-rich Lake Grosse Fuchskuhle (53°105774N,12°98454553E) located in northeastern Germany. Here, we briefly outline the ecologically relevant information from the genome of the freshwater *Sphingomonas* sp. strain FukuSWIS1 to obtain insights into their metabolic capabilities.

*Sphingomonas* sp. strain FukuSWIS1 genome sequencing was performed at the Berlin Center for Genomics in Biodiversity Research (BeGenDiv, http://www.begendiv.de/) using the Illumina MiSeq system following the 2×150-bp paired-end sequencing workflow. Genomic sequencing libraries were prepared using an Illumina TruSeq DNA sample preparation kit following the manufacturer’s instructions. Sequencing reads were *de novo* assembled using Velvet assembler (v1.2.10) (3) with k-mers of 63 for this genome. We generated 288 DNA scaffolds out of the shotgun assembly from the FukuSWIS1 genome, with *N*_50_ values of 30. The contigs of this genome were submitted to MG-RAST (4) and IMG-ER (5) for annotation and data analysis. This represents a draft genome size of 3.89 Mb, and the G+C content was found to be 65%. Strain FukuSWIS1 has 50 tRNA-coding genes and only one 5S rRNA gene, aside from one gene encoding for 16S rRNA and 23S rRNA. 16S rRNA gene sequence similarity was highest for the 16S rRNA gene sequence belonging to *Sphingomonas* sp. M3C203B-B obtained from Lake Vostok ice accretion in Antarctica (6).

The strain expresses photopigment BChl *a* under aerobic conditions and represents an orange-pigmented, rod-shaped Gram-negative alphaproteobacterium. Genes involved in the synthesis of the photosynthetic reaction centers (*pufBALM* genes) and bacteriochlorophyll *a* (*bchCXYZ* genes) biosynthesis are present in its genome, which suggest their capability to use light as an energy source. The presence of carotenoid genes (*crtZW-CYIB*) indicates the formation of beta-carotene, which potentially participates in the functionality of another photopigment coded in the genome, i.e., bacteriorhodopsin. Genes involved in autotrophic carbon fixation, however, are missing in the draft genome and might underline their photoheterotrophic character. This *Sphingomonas* strain possesses genes encoding for aromatic compound decomposition (like ring-cleavage extradiol dioxygenase-encoding and protocatechuate 3,4-dioxygenase-encoding genes) and harbor several genes coding for cell-wall degradation, e.g., cellulose 1,4-beta-cellobiosidase-, beta-xylosidase-, and xylanase-encoding genes. Therefore, humic-rich substrates in lakes may serve as a potential target for microbes to sustain their cellular metabolic processes. The presence of iron sequestering genes in its genome hints at their capability to counter iron-limited scenarios like in acidic and humic-matter-rich lake ecosystems. Overall, light energy not only seems to be directly harvested by *Sphingomonas* “light-harvesting-complexes,” but the photodegradation products of humic matter can also be channeled into the central metabolic pathway. A detailed genome comparison with other genomes (e.g., *Sphingomonas* sp. FukuSWIS6.2 from oligotrophic Lake Stechlin, Genbank accession number JPDP00000000) will shed more light upon the lifestyle exhibited by cosmopolitan *Sphingomonas* sp. strain FukuSWIS1.

### Nucleotide sequence accession number.

The whole-genome shotgun information for *Sphingomonas* sp. strain FukuSWIS1 was submitted to GenBank under the accession no. JPJC00000000.
